# Neural signature of the perceptual decision in the neural population responses of the inferior temporal cortex

**DOI:** 10.1038/s41598-022-12236-y

**Published:** 2022-05-23

**Authors:** Mohammad-Reza A. Dehaqani, Nazli Emadi, Abdol-Hossein Vahabie, Amin Zandvakili, Hossein Esteky

**Affiliations:** 1 Cognitive Systems Laboratory, Control and Intelligent Processing Center of Excellence (CIPCE), School of Electrical and Computer Engineering, College of Engineering, University of Tehran, Tehran, Iran; 2 School of Cognitive Sciences, Institute for Research in Fundamental Sciences, Tehran, Iran; 3 Research Center for Brain and Cognition, School of Medicine, Shahid Beheshti University of Medical Sciences, Tehran, Iran; 4 Department of Psychiatry and Human Behavior, Alpert Medical School of Brown University, Providence, RI 02906 USA; 5 Department for Brain Science and Technology, Pasargad Institute for Advanced Innovative Solutions (PIAIS), Tehran, Iran

**Keywords:** Neuroscience, Cognitive neuroscience

## Abstract

Rapid categorization of visual objects is critical for comprehending our complex visual world. The role of individual cortical neurons and neural populations in categorizing visual objects during passive vision has previously been studied. However, it is unclear whether and how perceptually guided behaviors affect the encoding of stimulus categories by neural population activity in the higher visual cortex. Here we studied the activity of the inferior temporal (IT) cortical neurons in macaque monkeys during both passive viewing and categorization of ambiguous body and object images. We found enhanced category information in the IT neural population activity during the correct, but not wrong, trials of the categorization task compared to the passive task. This encoding enhancement was task difficulty dependent with progressively larger values in trials with more ambiguous stimuli. Enhancement of IT neural population information for behaviorally relevant stimulus features suggests IT neural networks' involvement in perceptual decision-making behavior.

## Introduction

Classifying complex visual objects is a key cognitive function of primate brains. It has been shown that object category information is encoded by the activity of single inferior temporal (IT) cortex neurons. Specifically, neurons with selective responses to face and body have been found in the IT cortex of monkeys^1–5^. We have previously shown that electrical stimulation of face-selective neurons can bias monkeys' perceptual decision-making when categorizing ambiguous face/object stimuli^6^. However, object category information of ambiguous visual stimuli is only poorly encoded by the responses of IT single neurons^2,7^. When the visual cortex is challenged by noisy stimulus in object recognition tasks, the top-down attentional mechanism can enhance the category neural code in the IT cortex^2,8^. Specifically, the activity of IT single neurons is modulated by cognitive functions such as attention^9–11^ and memory^12–14^ regardless of the visual signal level. While so much has been revealed about the impact of behavioral tasks on sensory code at the IT single cell level, little is known about the potential impact of behavioral tasks on visual information in the IT neural population. Among the few studies that have examined IT neural population task dependent sensory encoding are experimental and theoretical works that show the effects of task demands on the responses of the IT cortex in color-selective neurons^15,16^. But IT cortex is mainly involved in object recognition, and task dependence of IT network activity in object recognition tasks is unclear.

Category information is represented by the pattern of neural activity in monkey and human visual areas^3,17^. The IT stimulus response selectively is enhanced during visual object categorization while the variability of single neural responses decreases^7^. A visual stimulus may be recognized by decoding the activity of single neurons or a population of neurons^3,4,18^. Traditional views suggest sparse coding by gnostic neurons^19^, but recent studies have pointed to the importance of population coding^3,20,21^. Population coding is more resilient to external noise (stimulus visibility) or the internal noise that arises from spontaneous or evoked activities of neurons unrelated to the task at hand^22^. Population coding of visual information has been reported in different visual areas such as V1^23^, MT^24^, V4^25^, and IT^3,20,21^. However, few studies have shown a link between neural population coding and behavior in visual areas^24,26^. Many studies used the choice probability to predict monkeys’ choice based on the firing rate of single neurons across visual steams^27–32^. However, it is not clear whether the object category information in the spiking activity of the IT neural population is related to object recognition and perceptual decision-making.

In the current study, we recorded single unit spiking activities of IT neurons in macaque monkeys performing passive viewing and two-alternative forced-choice body/non-body discrimination tasks. We applied population encoding and decoding analysis to explore task-dependent sensory enhancement as a function of task difficulty in IT neural population activity and examined the relationship between IT population code and behavioral choice. We found that enhancement of category information in the population of neural responses is associated with monkeys' correct choice. Interestingly, this behaviorally contingent neural activity modulation was task difficulty dependent, showing progressively larger enhancements in trials with more ambiguous stimuli. Even when neurons with no category selective responses were used for the population analysis, the population category information was present. Our results suggest an involvement of IT neural networks in perceptual decision-making behavior.

## Results

**Figure 1:**
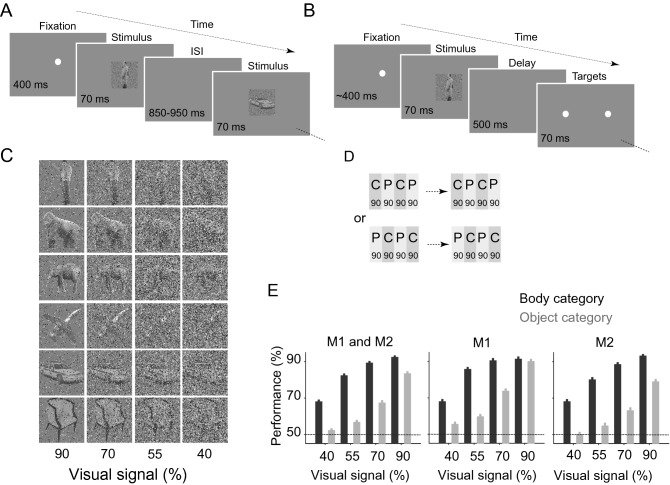
Task and performance. (**A**) Passive fixation task (ISI: inter-stimulus interval). (**B**) Body/object categorization task. (**C**) Exemplar body and object images in different noise levels. Numbers below the images show the percent of the added noise in each column of images. (**D**) Order of the tasks in a session. Each bar represents one block of 90 trials of categorization (C, dark gray) or passive (P, light gray) tasks. (**E**) Monkeys' performance in the categorization task was plotted for both (M1 and M2 subplot) and each monkey (M1 subplot and M2 subplot). The dashed line indicates 50% chance level. Error bars indicate the SEM.

Spiking activity of 123 single neurons was recorded from the inferior temporal cortex (IT) of two macaque monkeys under passive viewing and two-alternative forced-choice body/non-body categorization (Fig. 1A, B). The stimulus set was identical in the passive viewing and categorization tasks. The stimuli were images of bodies (human, monkey, four-leg; n = 90) and non-bodies (airplane, car, chair; n = 90)^7^. Each stimulus was presented with four levels of added noise to create various task difficulties (Fig. 1C). In each recording session, blocks of the passive and categorization tasks were presented in an interleaved order (Fig. 1D). As expected, monkeys' performance declined as the stimulus visibility decreased (Fig. 1E). A selectivity index (SI, see “Methods”) defined neural response category selectivity. Of 123 recorded single neurons, 75 and 48 were defined as body and non-body selective neurons, respectively (see “Methods”). We used body neurons for population analyses unless otherwise specified.

**Figure 2:**
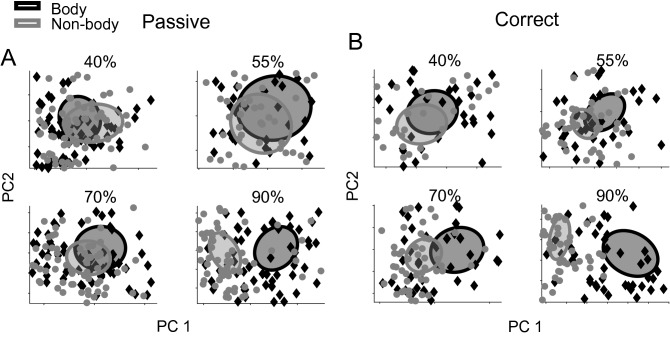
Low dimensional representation of subcategories in IT neural population activity. Representation of the categories in the first two most informative dimensions of the principal component analysis is shown for passive (**A**) and correct (**B**) trials. Ellipses demonstrate two standard deviations of the distribution of category members in the 2D representations. Diamonds and circles show body and non-body stimuli, respectively.

We used principal component analysis (PCA, see “Methods”) to visualize the distribution of category information represented in the IT neural population responses. In this analysis, the eigenvectors of the neural covariance matrix were used to make a transformation matrix from the high dimensional to the lower dimensional neural spaces. In the passive viewing task, we found that IT neural population responses differentiated body/object stimulus images of the most visible stimuli with a 90% signal level (Fig. 2A). We observed more distinction between body and non-body representation in reduced neural space for 90% signal level stimuli than other signal levels. We conducted the same analysis using the correct and wrong trials to assess the relationship between IT category representation and the monkeys' behavioral choice. The categorization information of IT neural population responses was enhanced in all of the stimulus signal levels only in the correct trials (Fig. 2B). On average, 64% ± 2% of the variance was captured by the first two dimensions of the PCA in passive and 67% ± 3% in active trials (the SEM computed across visual signals). The representation of the wrong trials in reduced neural space showed no clear body and non-body stimuli separation (Fig. S1).

**Figure 3:**
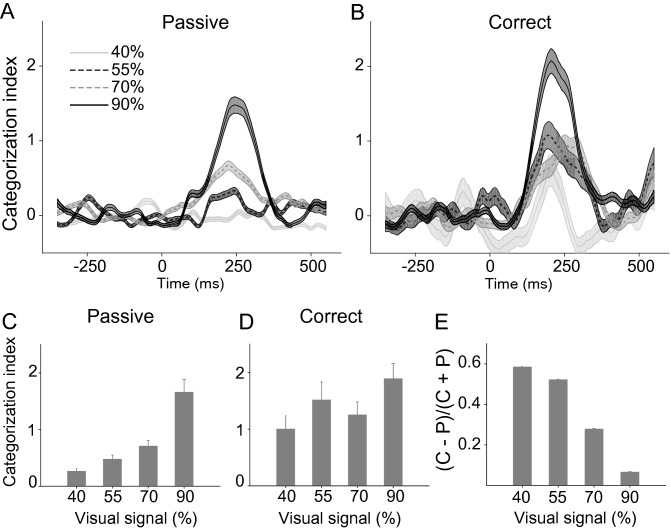
Category information of body-selective neural population in different stimulus noise levels and task conditions. Time course of category information in passive (**A**) and correct (**B**) trials in different signal levels. Data points are plotted in the middle of each bin. Bar graphs illustrate the CI during 150 to 350 ms after stimulus onset in passive (**C**) and correct (**D**) trials. (**E**) Category information in the correct trials compared to passive trials. Error bars represent the STD of bootstrap samples.

We introduced a categorization index (CI) to quantify the population-based category coding in the IT cortex (see “Methods”). This method can be applied to high dimensional datasets (here, 123 neurons) with a limited number of data points. To study the temporal dynamics of the category information across the IT neural population, we calculated CI values in 100-ms sliding windows with 5-ms steps for different task difficulties (Fig. 3A and B). The time courses were offset by subtracting the mean value of CI computed at [−400 0] ms interval from stimulus onset. As expected, lower CI values were observed in more noisy stimuli (CI values from 150 to 350 ms after the stimulus onset; these values were not offset: 90% = 1.66 ± 0.22; 70% = 0.71 ± 0.1; 55% = 0.48 ± 0.07; 40% = 0.26 ± 0.05; Fig. 3C). There was a strong correlation between the category information and the level of stimulus visibility (r = 0.95, P < 0.05). In the passive condition, the IT neuronal population did not convey significant information at 40% signal level (*p* = 0.75; the significance was checked by bootstrap confidence interval with the values of CI in [150 350] ms and [−200 0] ms time windows).

The temporal pattern of CI in the correct trials showed an enhancement in all signal levels (Fig. 3B). CI values measured during 150 to 350 ms time window revealed that category information was significantly enhanced in the correct trials compared with the passive trials (Fig. 3D; 40% = 1 ± 0.23, 55% = 1.51 ± 0.32, 70% = 1.25 ± 0.23, 90% = 1.89 ± 0.27; two-way ANOVA with task conditions (passive vs. correct) and noise levels as the two factors, *P*_*passive vs. correct*_ = 0.033, P_noise level_ = 0.0596; one replicate for each signal level). The enhancement of category information was similar in both monkeys (monkey 1: *P*_*passive vs. correct*_ = 0.007, P_noise level_ = 0.017; monkey2 *P*_*passive vs. correct*_ = 0.108, P_noise level_ = 0.088).

We calculated a normalized CI index to quantify the enhancement in the categorization information in the correct vs. passive conditions (see “Methods”). Interestingly, we found a more considerable enhancement of category information in more demanding trials with less stimulus visibility (Fig. 3E; Pearson correlation, r = −0.98, *P* < 0.01). These results show that while category information was absent in the noisiest condition in the passive trials, this information emerged in the response of IT neurons only in the correct trials of the categorization task. The main results were independently replicated in each monkey: enhancement of category information in 40% signal level was greater than 90% signal levels (Figure S2; bootstrap confidence interval; Monkey 1 and 2 *p* < 0.001).

**Figure 4:**
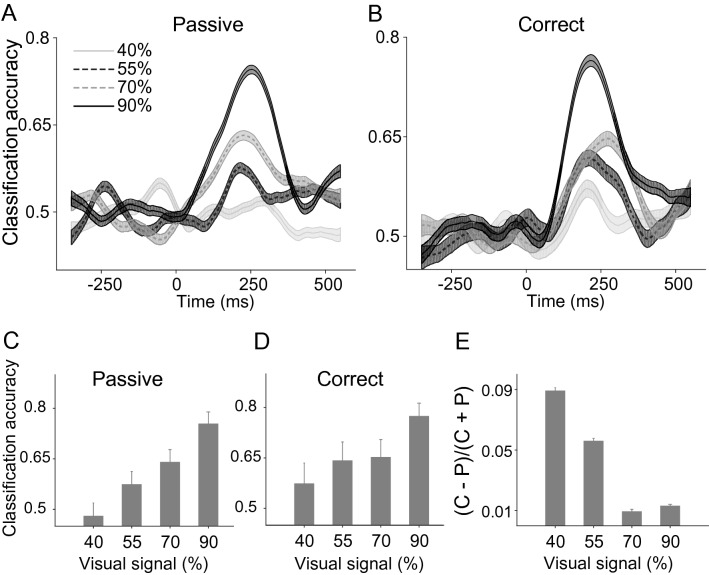
SVM classifier performance of body-selective neural population in different stimulus noise levels and task conditions. Time course of decoding accuracy in passive (**A**) and correct (**B**) trials in different signal levels. Data points are plotted in the middle of each bin. Bar graphs illustrate the classification accuracy during 150 to 350 ms after stimulus onset at different noise levels in passive (**A**) and correct (**B**) trials. (**E**) Classification performance in correct trials relative to passive trials. Error bars represent the STD of bootstrap samples.

We also tested the classification performance of IT neurons using the Support Vector Machine (SVM) classifier (see “Methods”). The classifier results were consistent with the PCA and CI results. There are similar temporal dynamics for representing category information at different task difficulties in correct and passive trials (Fig. 4A and B). In the passive condition, we found high performance for the low noise stimuli (90% signal; decoding accuracy = 0.75 ± 0.04) with systematically lower performances for more noisy stimuli leading to chance level performance for the highest noisy stimuli (40%) (Fig. 4C; 70% = 0.64 ± 0.04 , 55% = 0.57 ± 0.04 ;40% = 0.48 ± 0.04; Pearson correlation, r = 0.99, *P* < 0.001; using bootstrap interval shows that there is no significant difference between chance level (0.50) and decoding accuracy in 40% signal level; *p* = 0.3). Compared to the passive condition, classification accuracy increased in the correct trials, respectively (Fig. 4D: 90% = 0.77 ± 0.04; 70% = 0.65 ± 0.05; 55% = 0.64 ± 0.06; 40% = 0.57 ± 0.06). The results also confirmed a larger enhancement of the classification accuracy in more difficult trials (Fig. 4E, Pearson correlation, r = −0.9, *P* < 0.09). We observed statistically similar task dependency effects in SVM analyses (two-way ANOVA with task conditions (passive vs. correct) and noise levels as the two factors, *P*_*passive vs. correct*_ = 0.036, P_noise level_ = 0.011; one replicate for each signal level). Similar results were observed when data of each monkey was used separately (Fig. S3).

**Figure 5:**
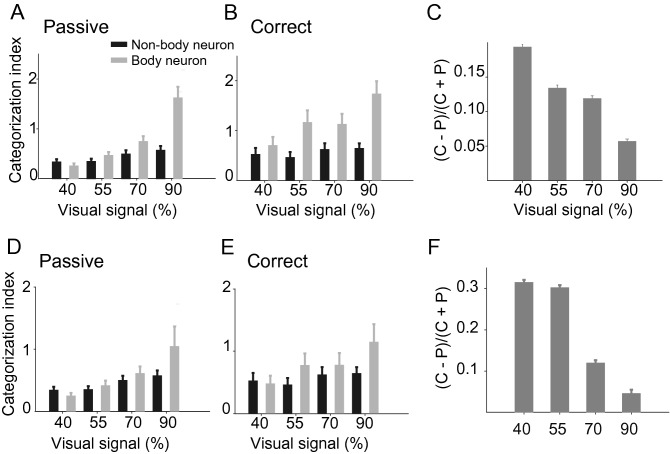
Comparison of category information conveyed by the body and non-body neurons. Mean category information from 150 to 350 ms after stimulus onset in passive (**A**) and correct (**B**) trials are shown for selective (body and non-body) and non-selective neurons. (**C**) Category information in the correct trials compared to passive trials in non-body neurons. The mean absolute value of SI in both body and non-body group was equalized. Mean category information from 150 to 350 ms after stimulus onset in passive (**D**) and correct (**E**) trials are shown for body and non‑body neurons. (**F**) The category information in the correct, compared to the passive, trials in SI-matched body neurons. Error bars represent the STD of bootstrap samples.

To this end, all of the analyses were performed using the responses of body neurons (SI > 0). We labeled 48 neurons that systematically gave more responses to non-body than body images (SI < 0) as non-body selective and used them to calculate population category information. We equalized the number of cells in the body and non-body groups by selecting 48 body neurons with the highest SI values to exclude the sample size bias. Body neurons conveyed significantly more category information than non-body neurons in both passive (Fig. 5A; ΔCI_body–nonbody_: 40% = -0.08 ± 0.071, *p* = 0.884; 55% = 0.119 ± 0.086, *p* < 0.001; 70% = 0.251 ± 0.129, *p* < 0.001; 90% = 1.051 ± 0.235, *p* < 0.001) and correct (Fig. 5B; ΔCI_body–nonbody_: 40% = 0.179 ± 0.213, *p* < 0.05; 55% = 0.705 ± 0.267, *p* < 0.001; 70% = 0.505 ± 0.24, *p* < 0.001; 90% = 1.093 ± 0.27, *p* < 0.001) trials. Similar to the body selective neurons, non-body neurons’ category information was also enhanced during the categorization of the more noisy stimuli (Fig. 5C; Pearson correlation; r = −0.98, *p* < 0.05). Although there was no significant difference in response, spike count (SC) of body and non-body neurons to all stimuli in passive and active trials (passive: SC_body-cells_: 1.82 ± 0.2, SC_nonbody-cells_: 2.03 ± 0.29, *p* = 0.64; active: SC_body-cells_: 1.9 ± 0.19, SC_nonbody-cells_: 2.32 ± 0.33, , *p* = 0.39) and task-dependent enhancement of category information in body selective neurons were more than two times larger than that of non-body selective neurons in the noisy stimuli (Δ[(C−P)/(C + P)]_body-nonbody_: 40% = 0.249 ± 0.0081, *p* < 0.001; 55% = 0.2927 ± 0.0072, *p* < 0.001; 70% = 0.0922 ± 0.0072, *p* < 0.001; 90% = −0.0233 ± 0.0067, *p* = 0.9). Consistent with CI in classification accuracy (CA), we observed that the body neurons conveyed significantly more category information than non-body neurons in both passive (ΔCA_body–nonbody_: 40% = −0.035 ± 0.058, *p* = 0.726; 55% = 0.053 ± 0.054, *p* < 0.05; 70% = 0.065 ± 0.059, *p* < 0.05; 90% = 0.168 ± 0.053, *p* < 0.001) and correct (ΔCA_body–nonbody_: 40% = 0.054 ± 0.082, *p* = 0.250; 55% = 0.163 ± 0.079, *p* < 0.001; 70% = 0.096 ± 0.067, *p* < 0.001; 90% = 0.164 ± 0.052, *p* < 0.001) trials.

To further study the relation of SI values of category representation in the non-body population, we equalized the mean absolute value of SI in both body and non-body groups and calculated the CI in both populations. The advantages of body neurons in category representation compared to non-body neurons was observed in both passive (Fig. 5D; ΔCI_body–nonbody_: 40% = −0.093 ± 0.072, *p* = 0.910; 55% = 0.063 ± 0.098, *p* = 0.248; 70% = 0.111 ± 0.133, *p* = 0.182; 90% = 0.471 ± 0.325, *p* < 0.001) and correct (Fig. 5E; ΔCI_body–nonbody_: 40% = −0.046 ± 0.185, *p* = 0.59; 55% = 0.314 ± 0.227, *p* < 0.01; 70% = 0.156 ± 0.234, *p* = 0.246; 90% = 0.504 ± 0.306, *p* < 0.001) trials. The SI-matched body neurons category information was also enhanced during the categorization of the more noisy stimuli (Fig. 5F; Pearson correlation; r = −0.95, *p* < 0.05).

We also measured IT neural category information in the wrong trials. We observed no significant category information in any of the signal levels using CI or SVM methods (ΔCI: 90% = 0.19 ± 0.48, *p* = 0.34; 70% = 0.24 ± 0.71 , *p* = 0.35; 55% = 1.01 ± 13.02, *p* = 0.47; 40% = 0.78 ± 3.57, *p* = 0.41—decoding accuracy: 90% = 0.57 ± 0.18, *p* = 0.27; 70% = 0.48 ± 0.15, *p* = 0.57; 55% = 0.52 ± 0.11, *p* = 0.43; 40% = 0.51 ± 0.08, *p* = 0.42). These analyses computed the difference of CI values in [150 350] ms and [−200 0] ms intervals. Significance was tested by the bootstrap confidence interval and comparing the values of CI in these two intervals. The significance of decoding bootstrap samples was tested against the chance level of the classifier (0.50). It should be noted that fewer wrong trials make the results noisier than the correct and passive trials.

**Figure 6:**
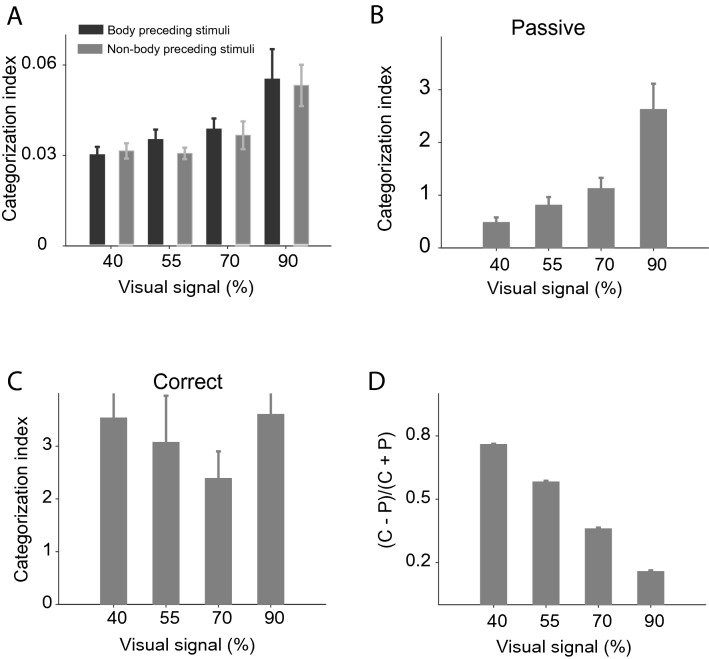
The potential impact of spike contamination evoked by preceding stimuli. The trials were divided into two groups for each neuron: trials in which body images preceded the stimulus and those in which a non-body image precedes the stimulus. (**A**) The mean values of CI in the passive condition from 150 to 350 ms after stimulus onset were plotted for each group, separately. Bar graphs illustrate the CI during 150 to 350 ms after stimulus onset in rate-matched populations in passive (**B**) and correct (**C**) trials. (**D**) Category information in the correct, compared to passive, trials during rate-matched population. Error bars represent the STD of bootstrap samples.

There was a shorter interstimulus interval in our study in the passive compared to the active condition. So there might be a higher chance of response contamination by preceding stimuli in the passive condition. To test this possibility, we divided trials into two groups for each neuron: trials in which a body image preceded the stimulus and those in which a non-body image preceded the stimulus (Fig. 6). We then calculated the CI values of neurons in each group. There was no significant difference in category representation between the two groups (Fig. 6A: (∆CI: 90% = 0.0021 ± 0.008, *p* = 0.78; 70% = 0.0021 ± 0.0061, *p* = 0.23; 55% = 0.0046 ± 0.0038, *p* = 0.30; 40% = 0.0013 ± 0.0037, *p* = 0.77, Wilcoxon’s signed-rank test, two-sided). Furthermore, we matched each neuron’s median responses in the passive and active trials to exclude the impact of rate magnitude. For each neuron in the active condition, we eliminated the stimulus with the highest response until there was no significant difference between passive and active neural response distributions (*p* > 0.05 using Wilcoxon’s signed-rank test, two-sided). Then we build the population’s matrix using the rate-matched neurons and computed the CI (Fig. 6B and C; Passive: 90% = 2.62 ± 0.49, 70% = 1.12 ± 0.2, 55% = 0.81 ± 0.15, 40% = 0.48 ± 0.1; Correct: 90% = 3.6 ± 0.7, 70% = 2.39 ± 0.51, 55% = 3.07 ± 0.88, 40% = 3.53 ± 1.08). The task dependent enhancement of category information was preserved in the median-matched conditions (Fig. 6D; r = −0.99; *p* = 0.003).

To separate the effect of task-dependent enhancement on category representation, we compare body and non-body stimulus representation (body and object categories) in correct compared to passive trials for the noisiest level stimuli (i.e., 40% signal level). We computed the decoding accuracy of the SVM classifier for the body and non-body stimuli using the confusion matrix. Greater enhancement of representation in the body compared to non-body stimuli was observed at the noisiest level of the signal in the correct trials (body category: 0.098 ± 0.004; object category: 0.081 ± 0.003; *p* < 10^−3^). Together, these results suggest that attention can contribute to enhancement of body representation in correct trails.

**Figure 7:**
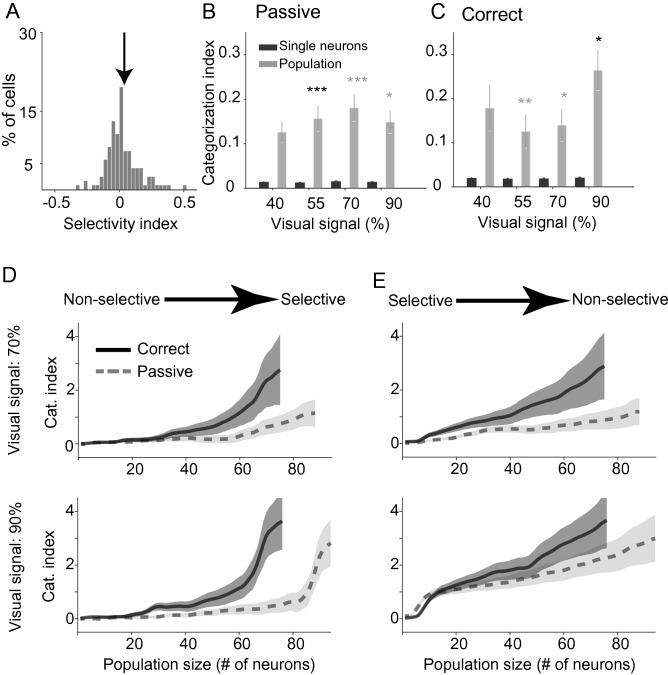
Comparison of category information conveyed by non-selective neurons computed for neural population and unit activities. The distribution of the selectivity index (**A**) is shown for all neurons. Arrow indicates the mean of selectivity index. Mean category information from 150 to 350 ms after stimulus onset calculated by CI in passive (**B**) and correct (**C**) trials are shown for the individual units and the neural population in non-selective neurons. Error bars represent the STD of bootstrap samples in population and SE in single neuron sub-plots. (**D**, **E**) Plots show the categorization index of the population of neurons as a function of the number of single units used for making the populations. Neurons were sorted based on SI, and sets of neural population were made by subsequently adding new units with increasing (**D**) and decreasing (**E**) SI values. Significance was computed with a bootstrap method to estimate 95%, 99%, and 99.9% confidence intervals. Asterisks indicate a significant difference between groups with different neural selectivity (*p < 0.05, **p < 0.01, ***p < 0.001).

Category information of individual neurons may affects our measurement of the neural population category code. To rule out this confounding effect, we calculated population category information using 36 neurons with SI values at, or close to, zero labeled as non-category selective (Fig. 7A; −0.037 < SI < 0.037). None of these neurons showed significant category information in the passive or active conditions (*p* < 0.05; 95% bootstrap confidence interval). By definition, there was no category information at a single cell level in the passive (Fig. 7B; 40% = 0.0142 ± 0.0029, *p* = 0.71; 55% = 0.0109 ± 0.0009, *p* = 0.35; 70% = 0.012 ± 0.002, *p* = 0.28; 90% = 0.0112 ± 0.0012, *p* = 0.20) or the correct (Fig. 7C; 40% = 0.0217 ± 0.0021, *p* = 0.71; 55% = 0.0204 ± 0.0038, *p* = 0.94; 70% = 0.0135 ± 0.0019, *p* = 0.09; 90% = 0.0181 ± 0.0032, *p* = 0.11) trials. Interestingly, non-selective neurons also conveyed significant category information at the population level in both the passive (Fig. 7B; 40% = 0.13 ± 0.02, *p* = 0.6810; 55% = 0.16 ± 0.03, *p* < 0.001; 70% = 0.18 ± 0.03, *p* < 0.01; 90% = 0.15 ± 0.03, *p* < 0.05) and the correct (Fig. 7C; 40% = 0.18 ± 0.05, *p* = 0.76; 55% = 0.13 ± 0.04, *p* =  < 0.01; 70% = 0.14 ± 0.04, *p* =  < 0.05; 90% = 0.26 ± 0.05, *p* < 0.05) trials.

To examine the contribution of a single unit in the observed population phenomena, we sorted the IT neurons based on their SI (see “Methods”). Then we made a set of the neural population by adding new single units (with increasing, Fig. 7D, and decreasing SI, Fig. 7E). Using this approach, we computed the CI as a function of the number of neurons in different conditions. In both non-selective (Fig. 7D) and selective (Fig. 7E) populations the correct trials conveyed more category information for a given number of neurons (for example the population of 50-neurons, ΔCI in non-selective populations: 70% = 0.48 ± 0.33, *p* ≤ 0.01; 90% = 0.54 ± 0.3, *p* < 0.001, and ΔCI in selective populations: 70% = 0.95 ± 0.5, *p* < 0.001; 90% = 0.59 ± 0.56, *p* < 0.01).

**Figure 8:**
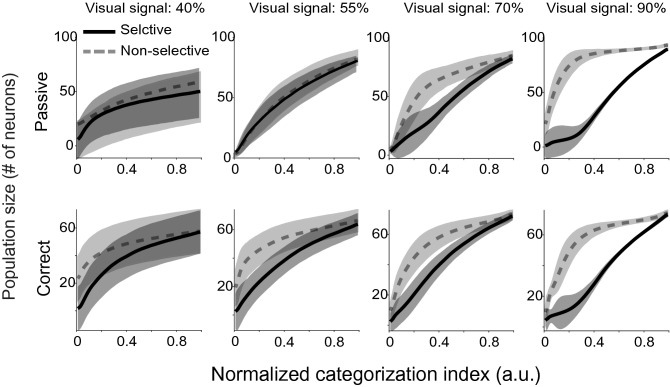
The number of single units as a function of normalized categorization index. The estimated number of units (y-axis) that can explain X% of the ultimate CI (x-axis) is plotted in each condition. Columns show visual signals and the two rows depict passive and correct trails. Shaded areas represent the STD of bootstrap samples.

To understand how many neurons are needed to sufficiently represent category information in the population responses, we estimated the number of units explaining the maximum CI in each condition. Figure 8 shows the number of single units that can sufficiently explain each normalized CI value in the passive and active conditions. Figure 8 illustrated that in low noise stimuli, the small number of the most informative single units accounted for the most category coding. Table 1 lists the number of single units that can explain 25%, 50%, 75%, and 95% of the ultimate CI in the passive and correct conditions (rows) for different stimulus noise groups for selective and non-selective neural populations (column). More non-selective neurons should be recruited to represent less informative stimuli (Table 1).

**Table 1:** The number of single units that can explain 25%, 50%, 75%, and 95% of the maximum CI.

	Selective				Non-selective			
	VS = 40%	VS = 55%	VS = 70%	VS = 90%	VS = 40%	VS = 55%	VS = 70%	VS = 90%
**0.25% of CI**								
Passive	31.8 ± 30	37.1 ± 12.1	23.1 ± 7	10.1 ± 5.8	35.3 ± 20	36.4 ± 12.4	53.5 ± 14.5	79.3 ± 8.6
Correct	30.2 ± 19.4	27.4 ± 11.4	25.7 ± 10.1	14 ± 4.7	43.9 ± 16.3	48.4 ± 10.5	49.1 ± 9.5	54.7 ± 7.7
**0.50% of CI**								
Passive	40.5 ± 32.1	57.4 ± 12.9	46.9 ± 17.3	42.6 ± 16.8	45.8 ± 21.8	58 ± 11.8	69 ± 8.1	87.2 ± 2.2
Correct	44.1 ± 18.3	44.4 ± 13.8	47.5 ± 11.9	38.9 ± 11.8	50.6 ± 15.9	57 ± 9.2	61.7 ± 6.2	65.5 ± 2.6
**0.75% of CI**								
Passive	45.6 ± 33.4	71.7 ± 11.4	67.8 ± 16.4	71.1 ± 12.9	53.4 ± 22.5	72.5 ± 9.7	77.7 ± 6.3	89.7 ± 0.9
Correct	51.4 ± 16.4	55.6 ± 12.2	61.9 ± 9.3	58.7 ± 7.9	54.7 ± 15.7	61.9 ± 8.4	67.6 ± 4	69.2 ± 1.3
**0.95% of CI**								
Passive	49.4 ± 34.2	80.6 ± 9	80.2 ± 12	86.8 ± 7.9	58.2 ± 22.9	81 ± 7	83.7 ± 4.1	92.4 ± 1.1
Correct	56.2 ± 15	62.4 ± 10.2	70.4 ± 5.8	71 ± 4.9	57.3 ± 15.7	65.6 ± 7.8	71.9 ± 3.4	73 ± 1.7

The table list the number of single units that can explain X% of the ultimate CI in the passive and correct conditions (rows) for different noisy stimuli of the selective and non-selective neural populations (column). STD was estimated by bootstrap.

Body selectivity in the IT cortex has been reported using electrophysiological recordings of neuronal spiking activities^3,33^ and functional MRI^34–36^. The presence of MRI-defined body selective regions suggests clustering of body selective neurons in the IT cortex. To examine the relation of IT neurons' body selectivity and location, we computed SI using the highest signal level and plotted SI values versus the anterior–posterior (AP) positions of the recorded neurons (Figure S4A and B). These plots illustrate the concentration of high-value SI neurons (values SI > 0.25) located within AP 14–18 mm in both monkeys.

We then used the high-valued SI neurons to construct the neural population and to compute category information. Figure S5 shows that similar task-dependent category information was observed during the categorization of the more noisy stimuli (CI_passive_: 40% = 0.05 ± 0.01, 55% = 0.08 ± 0.02, 70% = 0.15 ± 0.03, 90% = 0.77 ± 0.09; CI_correct_: 40% = 0.14 ± 0.05, 55% = 0.26 ± 0.06, 70% = 0.41 ± 0.08, 90% = 0.9 ± 0.12). Similar to neural population, a larger enhancement of category coding was observed in more ambiguous trials ((C−P)/(C + P): 40% = 0.456 ± 0.008, 55% = 0.546 ± 0.005, 70% = 0.477 ± 0.004, 90% = 0.082 ± 0.004).

**Figure 9:**
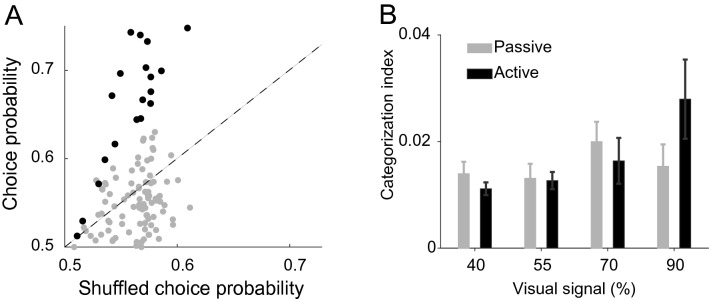
Choice probability computed for full noise stimuli. (**A**) Comparison of the CP and mean of shuffled CP for all IT neurons. The solid dots show neurons with significantly high CP values. (**B**) Bar plots show the mean value of CI during 150 to 350 ms after stimulus onset for neurons with high CP values in the passive and active trials. Error bars represent the SE of means in individual neurons.

To measure how well the firing rate of individual IT neurons predicted the behavioral responses of the monkey, we applied the area under ROC analysis. We computed choice probability (CP) using full noise stimuli for each neuron. In Fig. 9A, the neuron’s CP values are compared with the mean of the shuffled distribution. Neurons depicted with solid dots (n = 20) took values outside the 90% of the permuted distribution (CP = 0.67 ± 0.02). The category information coding of passive condition was compared with the correct trials in neurons with high CP (Fig. 9B; CI_passive_: 40% = 0.014 ± 0.01, 55% = 0.013 ± 0.012, 70% = 0.02 ± 0.017, 90% = 0.015 ± 0.018; CI_active_: 40% 0.011 ± 0.005, 55% = 0.013 ± 0.007, 70% = 0.016 ± 0.019, 90% = 0.028 ± 0.033). The CI was significantly higher for the correct, compared with the passive, trials in stimuli with high visual signals (∆CI = 0.013 ± 0.018; *p* = 0.005, Wilcoxon’s signed-rank test, two-sided). The difference between the high-valued CP population and the population of all neurons suggest a difference between the neural mechanisms involved in choice representation and object representation.

## Discussion

The involvement of higher sensory areas in the neural processes related to perceptual decision-making has been extensively debated. Practically, all related studies have used responses of single units in the prefrontal cortex and different sensory areas to address this important question. Here, we have provided evidence that IT neural population responses convey more accurate body category information in the correct, but not wrong, trials of active categorization task compared with the passive vision condition. As expected, we found a larger amount of category information in the passive viewing condition as stimulus ambiguity decreased, confirming a faithful representation of the category boundary map at the neural population level. More importantly, we found enhancement of category information in the body selective neurons during the correct, but not wrong, trials of the categorization task. The response modulation in the correct trials was task difficulty dependent, showing progressively larger degrees of enhancement in trials with more ambiguous stimuli. Monkeys' wrong choices were correlated with a decline in IT neural population category information. Qualitatively similar results were observed for non-body neurons, but the category representation was significantly larger in the population of neurons with selective responses to the body. Critically, population category information was present even in responses of neurons that showed no category selectivity. These findings suggest that the IT cortex is involved in the discrimination of task-relevant category boundary map of familiar objects and might be part of a global decision-making network.

Theoretical studies of neural population coding have suggested a role for ensemble code in perception and decision making^37–40^. However, very few experimental studies have examined the link between neural population activity and behavior^20,41^. Here we show that category information in the neural population is task-dependent and enhances during object recognition compared to passive viewing tasks. Enhancement of category information was particularly observed when the basic population code, measured using the neural activity during the passive viewing condition, was insufficient to support reliable decisions due to high ambiguity in the visual stimulus. We also observed significantly higher levels of category information in the correct compared to the wrong trials showing a strong correlation between IT neural population code and monkeys' behavioral choice. Our results are consistent with other reports of the contribution of population-level representation to cognitive functions such as categorization, visual attention, and decision making^18,21,42–46^.

Ensemble of body selective neurons conveyed the highest amount of category information, and its category map underwent the highest degree of change in active vs. passive task. Non-body selective neurons showed a lower categorization index (CI) and enhancement of category boundary map in the active condition. These findings indicate a greater but not exclusive role for selective neurons in population coding. The highest values of CI during active conditions provide evidence for modulation of category representation by feedback signals. It has been shown that attention changes the responses of sensory neurons when performing a cognitive task^47–49^. Therefore, attention as one of the main components of perceptual decision tasks can contribute to this modulation.

It has been shown that the performance of highly direction-selective medial temporal neurons correlates with behavior better than the general neural population^24^. Another study has shown that detection of direction of visual motion mainly relies on the relative activity of neurons preferring opposite directions of motion^50^. Decoding mechanisms based on single neurons' information rely on the activity of the most informative units. In contrast, mechanisms that rely on a comparative analysis of the activation pattern across a neural population may use information from the whole population regardless of the stimulus selectivity of the constituent neurons^51^. We found that category selectivity of body neurons was enhanced at least three times more than those of non-body neurons. However, non-body neurons category selectivity was also significantly enhanced in the correct vs. passive condition. These findings suggest that the whole population of IT neurons may be used in behavioral choice and may be an integrated part of a global perceptual-contingent behavioral decision making.

Many studies show the importance of population-level representation for various cognitive functions and demonstrate that neural ensemble can utilize the non-selective neurons to improve coding^3,18,21,33,42,44,52,53^. Although simultaneous recording is necessary to capture some aspects of the population code, the enhanced population-level representation is mainly determined by increasing the dimension of neural space and inter-neuronal correlation^52^. For a single neural response space, the neural representation of two categories may fall within a single plane and cannot be read out using a simple mechanism. However, two categories can be linearly distinguishable when more neurons are added to high dimensional representations. Higher dimensionality plays an important role in the linear readout of category information. Although an enhancement in noise correlation can improve the coding ability (e.g., if signal and noise correlations were in a different orientation), the theoretical and experimental studies^52,54,55^ have shown that noise correlation can limit the amount of information coding in a neural population. Since our unit recordings were not simultaneous, we cannot address this limitation.

In summary, we have shown that enhanced category information in IT neural ensembles is associated with the correct categorization of visual objects. We observed greater enhancement of category coding in more difficult trials, and there was no significant category information in any of the signal levels during the wrong condition. This association between IT neural population activity and behavior indicates that the IT cortex is an integrated part of the global neural network responsible for perceptual decision making.

## Methods

### Subjects

Two male adult macaque monkeys (Macaca mulatta) were used in this study. Head restraints and recording chambers were stereotaxically implanted under aseptic conditions on the dorsal surface of the skull of the monkeys while the animals were anesthetized with sodium pentobarbital. The anesthesia was performed under ARRIVE guidelines. All experimental procedures were approved by the animal care and use committee of the Institute for Research in Fundamental Sciences (04-11-6415-2008). All methods were performed in accordance with the relevant guidelines and regulations.

### Stimuli

The stimuli were presented on a 19 inch CRT computer monitor placed 57 cm in front of the monkey seated in a primate chair. The stimuli were 7° × 7° in size grayscale photographs of bodies (including human, monkey, and four-leg) and objects (including aircraft, cars, and chairs). There were 90 images in each category (30 images per subcategory). Each stimulus was presented in four different signal levels. Each signal level was generated by assigning a uniformly distributed grayscale value to X% of image pixels, where 100-X was the absolute signal level and had one of the values of 90, 70, 55, or 40. These 720 noisy stimuli (2 categories, 90 stimuli in each category, 4 signal levels) and 90 full noise images (0% visual signal) were randomly presented to the monkeys without repetition.

### Behavioral paradigms

In each recording session, monkeys performed two tasks; passive fixation (passive) and two-alternative forced-choice body/non-body categorization (active) tasks. At the beginning of a recording session, 810 noisy stimuli in the image set were randomly divided into 9 blocks of 90 images separately for each task. Monkeys were presented with an interleaved order of passive and active blocks, starting randomly with any of them in each recording session. No cue was provided to the monkeys about the task change beforehand.

In the passive fixation task following 400 ms of fixation on a fixation point at the center of the screen, a random sequence of 90 images (7° × 7° in size) were presented to the monkey. Each image was presented once and for 70 ms with three variable blank intervals (850, 900, and 950 ms) between images. The monkey was rewarded with a drop of apple juice every 1.5–2 s as long as its gaze was maintained within a 2.4° × 2.4° fixation window at the center of the screen. The sequence stopped when the monkey broke the gaze fixation, and the fixation point reappeared after a 1500 ms blank interval.

The monkey started an active trial by fixating on a fixation point at the center of the screen for one of the three randomly selected durations (350, 400, or 450 ms) followed by a noisy image (7° × 7° in size) presented for 70 ms at the center of the screen. The images were presented in a pseudo-random order, assuring each image was only presented once. The image presentation was followed by a 500 ms blank interval followed by two small response targets presented 10 visual degrees to the left and right of the screen center. The left and right targets represented the body and non-body responses, respectively for one monkey and the opposite for the other one. Monkeys were required to make a saccade to the correct target no later than 300 ms after the onset of targets and maintain their gaze within a 2.4° × 2.4° fixation window on the saccade point for 150 ms. Correct responses were rewarded by a drop of apple juice. For full noise stimuli (0% visual signal), the monkey was rewarded randomly with a probability of 0.5. The inter-trial delay was 750 ms on the correct trials and 1500 ms on the false trials. An infrared eye-tracking system measured the eye position (i_rec, http://staff.aist.go.jp/k.matsuda/eye/).

### Recording

We recorded the spiking activity of 123 single units in the IT cortex of behaving monkeys (n = 49 in monkey 1 and n = 74 in monkey 2). For each neuron, data were collected during both active and passive tasks. In each recording session, tungsten electrodes (FHC, USA) were inserted into the IT cortex. The electrodes were advanced with an Evarts-type manipulator (Narishige, Japan) from the dorsal surface of the brain through a stainless steel guide tube inserted into the brain down to 10–15 mm above the recording sites. The recording positions were defined by the MRI images acquired before the surgery. Recordings were made on an evenly spaced grid, with 1-mm intervals between penetrations over a wide region of the lower bank of STS and TE cortices (12 to 18 mm and 13 to 20 mm anterior to the interauricular line in monkey 1 and monkey 2, respectively). The action potentials of single units were isolated in real-time by a template matching algorithm^56^. After isolating single units, monkeys were required to perform the passive fixation and the categorization tasks.

### Data analysis

Based on the similar trend of monkeys' behavior and a similar pattern in other results, data from two monkeys were combined in all analyses.

### Selectivity Index (SI)

The degree of category selectivity of each neuron for body versus object images was measured by SI:

$$ SI = \frac{\mu (B) - \mu (O)}{{\mu (B) + \mu (O)}} $$

μ(B) and μ(O) were the means evoked response of each neuron (within 70 ms to 420 ms after the stimulus onset) to body and object images, respectively. In each neuron, SI values were averaged across all signal levels in correct trials of the active task. Neurons with SI values larger than zero were considered as body selective.

### Population analysis

The individual neurons used in population analysis were recorded in different recording sessions with slightly different trial numbers (median = 810, mean ± s.e.m. = 791 ± 14). Neural subpopulations were randomly generated from the recorded neurons in each specific condition. Trial numbers between different neurons were matched by the mean number of body and non-body trials; by condition, we refer to the task and signal level. All of the neurons which have a trial number more than the mean number of trials in each category and condition were included in a population analysis (for all conditions: neuron number > 68; stimulus number > 86). Therefore we did not repeat any trial to make population. We equalized the number of body and non-body stimuli to prevent potential bias of stimulus number in each category. We randomly sampled the trials from the body and non-body stimuli for each neuron. The selected trials of N neurons were concatenated together to make the population response (R^N^ space). Each stimulus (x) is represented as a point in the R^N^ space $x \in R^{N}$, N is the number of neurons. We repeated sampling and concatenation 1000 times to provide a bootstrap procedure. Using this bootstrap method, we calculated population measurement distributions and estimated confidence intervals. The standard error (SE) was then estimated as the standard deviation of computed measurements over 1000 bootstrap runs.

### PCA

To illustrate categories represented in the population of neural response, we used Principle Component Analysis (PCA) to reduce the dimension of neural space and show the categories in two-dimension space^57,58^. The neural response from 150 to 350 ms after the stimulus onset was used in this analysis. The first two components with the greatest variance were used to generate the two-dimensional representation of the neural population space. To illustrate the body and non-body stimuli representation in reduced PCA space, we used the covariance matrix of stimuli in body and non-body categories. This matrix was illustrated by the ellipses that demonstrate two standard deviations of the distribution of category members in the 2D representations.

### Categorization Index

A categorization index was defined to quantify category information in the neural population. This index is based on the ratio of the between-category to within-category response variability. A Scatter matrix is a statistic used to estimate the covariance matrix of a high-dimensional space^59^. Scatters of the within-category subgroups (e.g., human and monkey body) and between-category subgroups (e.g., human body and car) were generated. The ratio of between-category scatters to the within-category scatter indicates the category information in the pattern of IT neural responses.

CI was measured in a 200-ms window from 150 to 350 ms after the stimulus onset in three steps:

First, we computed the center of mass of each category in R^N^ and also the mean across all categories, total mean:

$$ Mean\;vector\;of\;ith\;category\quad m_{i} = \frac{1}{{n_{i} }}\sum\limits_{{x \in C_{i} }} x $$

$$ Total\;mean\;vector\quad m = \frac{1}{n}\sum\limits_{i = 1}^{c} {n_{i} m_{i} } $$

Each image (x) is a point in the R^N^ space $x \in R^{N}$, *N* is the number of neurons and $C_{i}$ is the set of images in the *i*th category. *n*_*i*_ is the number of images in the *i*th category and *n* is the total number of images; *c* is the number of categories.

Second, the within and between scatters were computed with the following formulas:

$$ Scatter\, \, matrix\; \, for\;ith \, \;category\quad S_{i} = \sum\limits_{{x \in C_{i} }} {(x - m_{i} )(x - m_{i} )^{t} } $$

$$ Within - category\;scatter\; \, matrix\;S_{W} = \sum\limits_{i = 1}^{c} {S_{i} } $$

$$ Between - category\;scatter\;matrix\;S_{B} = \sum\limits_{i = 1}^{c} {n_{i} (m_{i} - m)(m_{i} - m)^{t} } $$

In within-category scatter matrix, the covariance matrix measures potential relations of the neural responses in a specific category. In between-category scatter matrix, the covariance matrix of the mean responses in each category and the total mean was computed.

Third, CI was computed as:

$$ CI = \sum\limits_{i = 1}^{n} {\lambda_{i} } $$

which $\lambda_{i}$ i = 1,2,.., n (n = number of neurons in population) are the Eigen valuse of $S_{W}^{ - 1} S_{B}$. This method of CI calculating can be applied to high dimensional datasets with a limited number of data points^33^. It is closely related to Fisher's information^52,59^, and the use of ANOVA in low dimensional datasets^60^. To calculate the standard error of the separability index, we used a bootstrapping process^61^. All of the calculations were repeated 1000 times on a random selection of stimuli. We used the standard deviation of bootstrap samples to compute confidence intervals and significances by checking whether the zero lay outside the confidence interval of different distributions or not. We applied the percentile-based method for statistical tests and the SE of bootstrap samples used for plotting error bars in the plots. We computed the p-values by counting the number of values that exceed the observed value. For comparing two bootstrap distribution. For measuring CI in time, 100 ms windows with 5-ms steps were used.

The CI in one-dimensional space is similar to F-statistic. So to study the amount of category information conveyed by every single neuron, we used CI computed for single neuron responses in a 200 ms window from 150 to 350 ms after the stimulus onset.

Significance of CI for population analysis in [150 350] ms time window was tested by bootstrap confidence interval and comparing the values of CI in this time window with the CI values computed in a 200-ms window before stimulus onset ([−200 0] ms). We used the Wilcoxon sign rank test to examine the significance of CI values for single cells in these two-time intervals.

Category information in correct trials comparative to passive trials was measured by the following formula using normalized values:

$$ \left( {{\text{CI}}_{{{\text{correct}}}} - {\text{ CI}}_{{{\text{passive}}}} } \right)/\left( {{\text{CI}}_{{{\text{correct}}}} + {\text{ CI}}_{{{\text{passive}}}} } \right) $$

#### Classifier

A decoding approach was used to measure the categorical information in the neural population. We trained a Support Vector Machine (SVM) classifier with a linear kernel^62^ on the population of neural response. We applied a fivefold cross-validation procedure to estimate the classifier's performance in the population in a 200-ms window from 150 to 350 ms after the stimulus onset. To generate the SEs of the classifier output (decoding accuracy), we used a bootstrap method and repeated the calculations 1000 times. In each repetition, the classification accuracy was computed cross-validation. We used the standard deviation of bootstrap samples to compute confidence intervals and significances. One hundred-ms windows with 5-ms steps were used to measure classification accuracy in time. Classification accuracy (CA) in correct trials compared to passive trials was measured by the following formula using normalized values:

$$ \left( {{\text{CA}}_{{{\text{correct}}}} - {\text{ CA}}_{{{\text{passive}}}} } \right)/\left( {{\text{CA}}_{{{\text{correct}}}} + {\text{ CA}}_{{{\text{passive}}}} } \right) $$

#### Choice probability

To quantify the relationship between neural response and choice of the monkeys, we computed empirical receiver operating characteristic (ROC) curves^28,63^ and used the area under curves as a measure of choice probability (CP). Based on the monkey’s category choice, the neural responses to full noise stimuli were divided into body and non-body distributions. The area under ROC gives a nonparametric and reliable measure for the separation of neural response evoked by monkeys’ decisions. Choice probability measured by ROC analysis represents the proportion of trials that could correctly predict the monkeys’ choice based on the firing rate of a single IT neuron. A value of 0.5 represents chance performance, and a value of 1 represents a perfect association between neural and behavioral responses. Statistical significance for the choice probabilities was computed using a permutation test^64^ with 1000 permutations. We calculated the CP for neural data after randomly shuffling the monkey’s choice. We obtained the chance distribution, and the actual ROCs that lay outside the confidence interval of the permuted distribution were considered significant (i.e., one-tailed test).

## Supplementary Information

Supplementary Information.
