# Supplementary material for: Neural signature of the perceptual decision in the neural population responses of the inferior temporal cortex
Source: Sci Rep. 2022 May 23;12:8628. doi: 10.1038/s41598-022-12236-y (PMC9127116; doi:10.1038/s41598-022-12236-y)
Supplement: Supplementary file 1 — Supplementary Information. [file 41598_2022_12236_MOESM1_ESM.pdf]

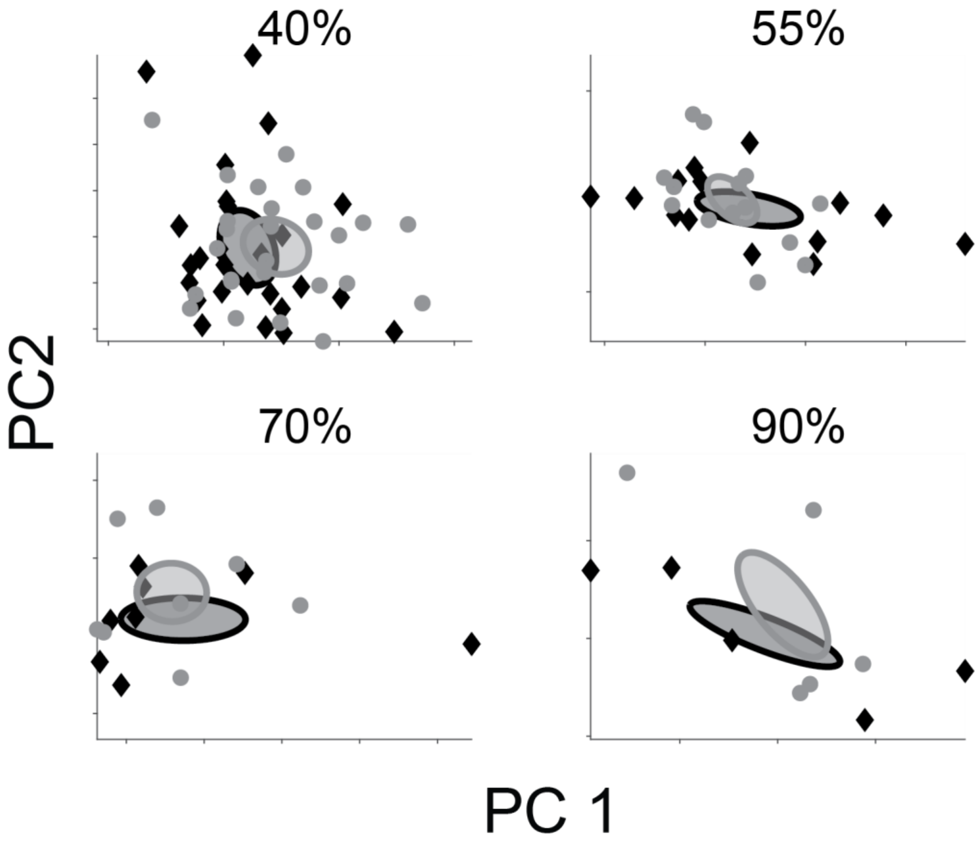

Figure S1: Low dimensional representation of subcategories in IT neural population activity.

Representation of the categories in the first two dimensions of principal component analysis is shown for wrong trials. Ellipses demonstrate two standard deviations of the distribution of category members in the 2D representations. Circles and diamonds showed the non-body and body stimuli, respectively.

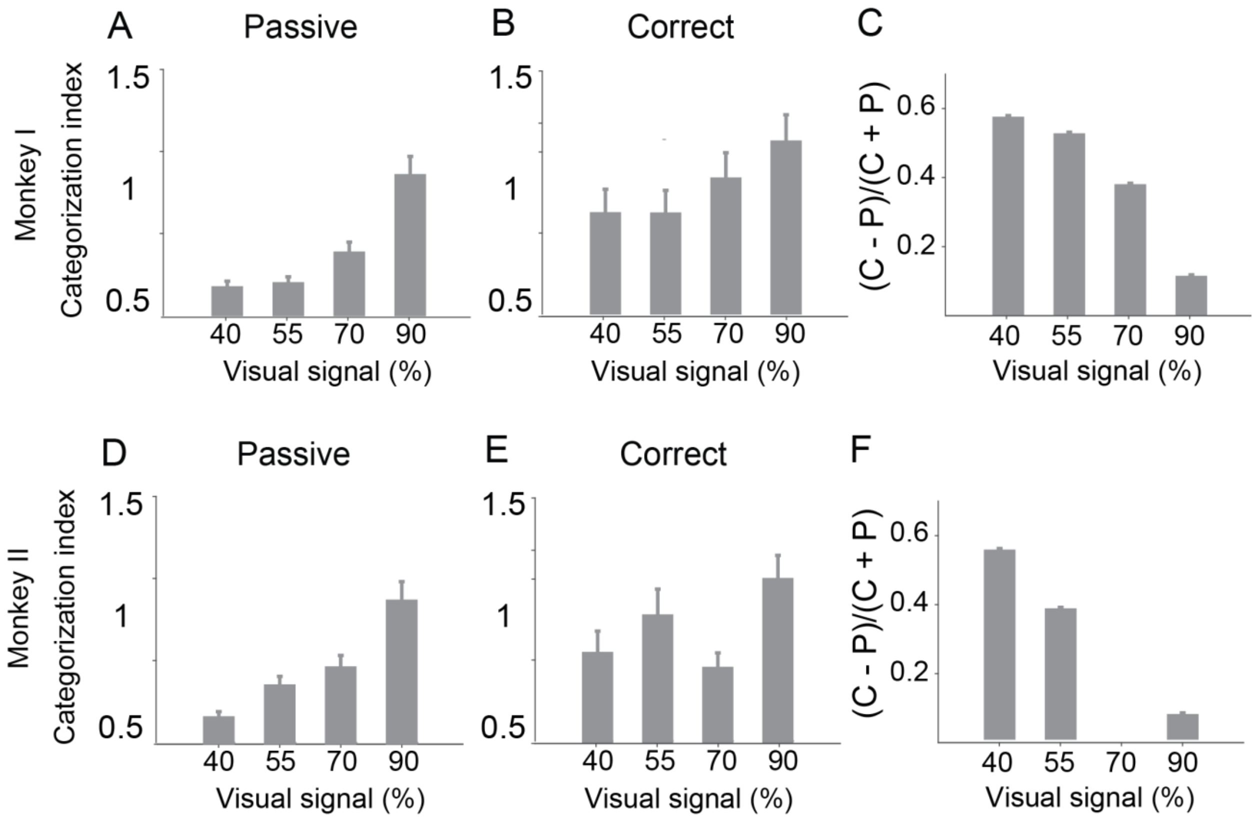

Figure S2. Category information in different stimulus noise levels and task conditions.

The CI was computed during 150 to 350 ms after stimulus onset for both monkeys. Category information in the correct trials compares to passive trials for Monkey I (A, B, and C) and Monkey II (D, E, and F). Error bars represent the STD of bootstrap samples.

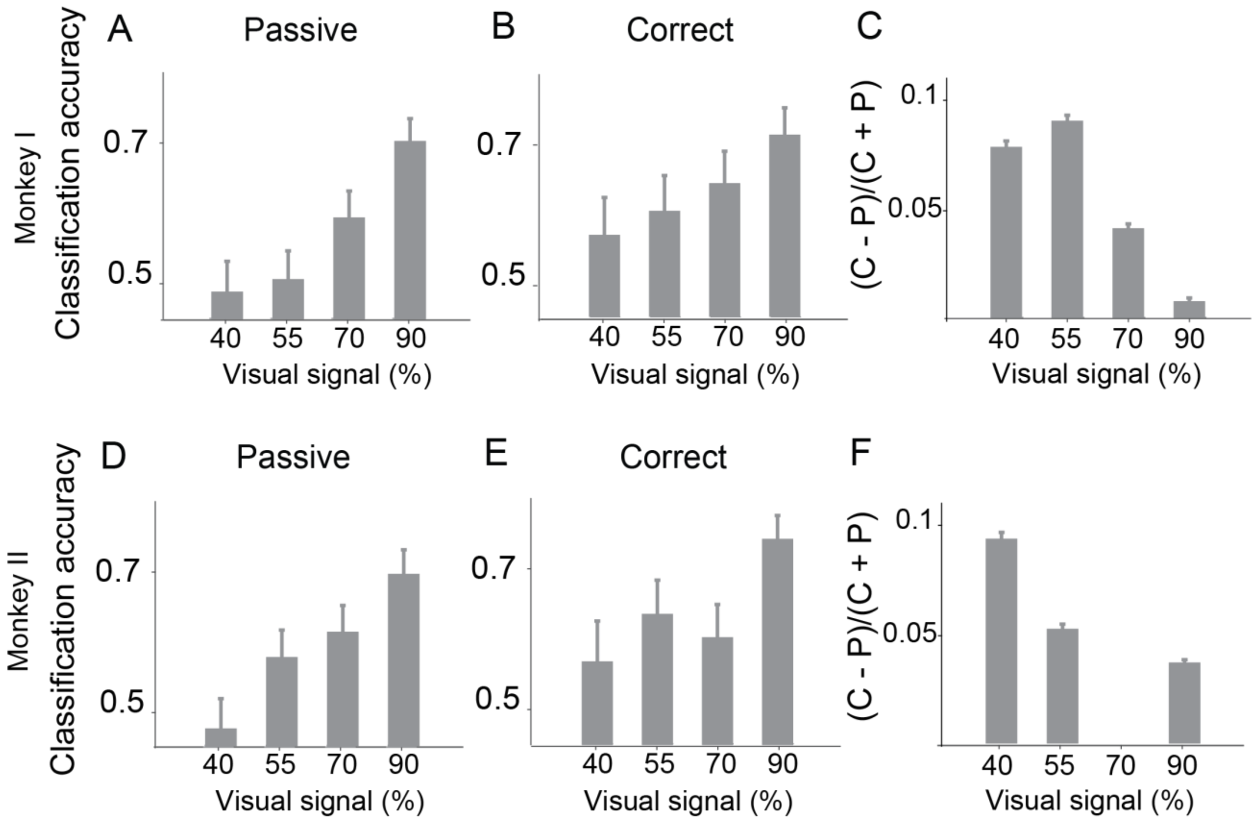

Figure S3. SVM classifier performance in different stimulus noise levels and task conditions.

The classification accuracy was computed during 150 to 350 ms after stimulus onset for both monkeys. The SVM performance in the correct trials compares to passive trials for Monkey I (A, B, and C) and Monkey II (D, E, and F). Error bars represent the STD of bootstrap samples.

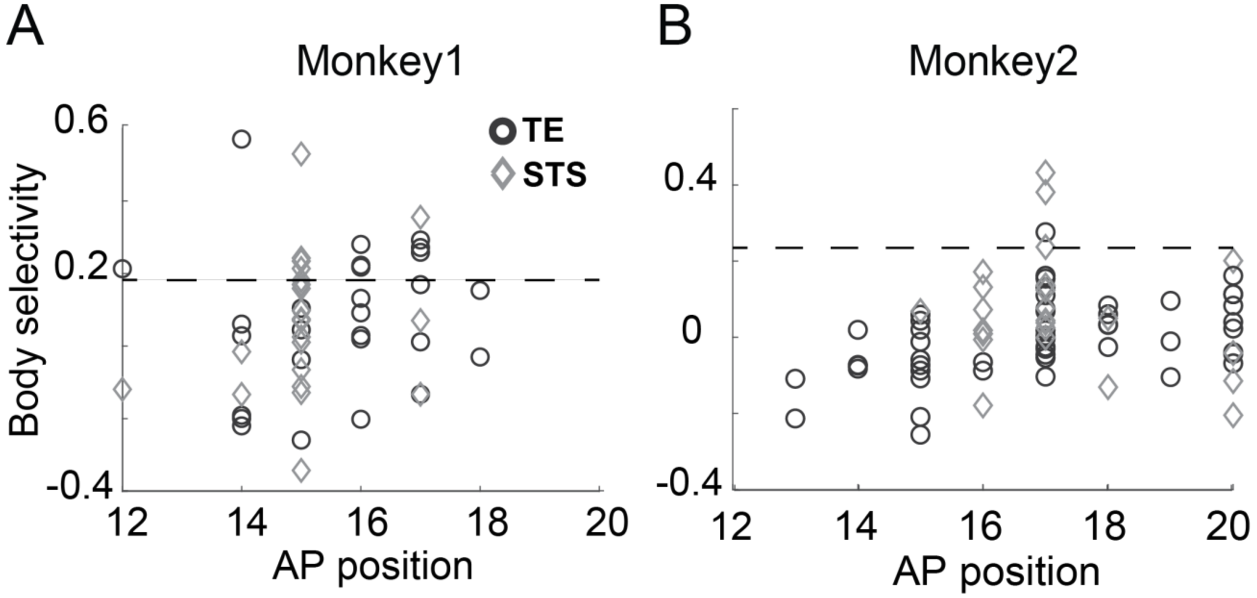

Figure S4. Relation of neurons' body selectivity and location of the recorded cells. The scatters demonstrate SI values versus the anterior-posterior (AP) positions for Monkey1 (A) and Monkey2 (B). Each point in the scatters shows one recorded neuron. The TE and STS neurons are labeled by circle and diamond, respectively and dashed line shows the 90th percentile of SI.

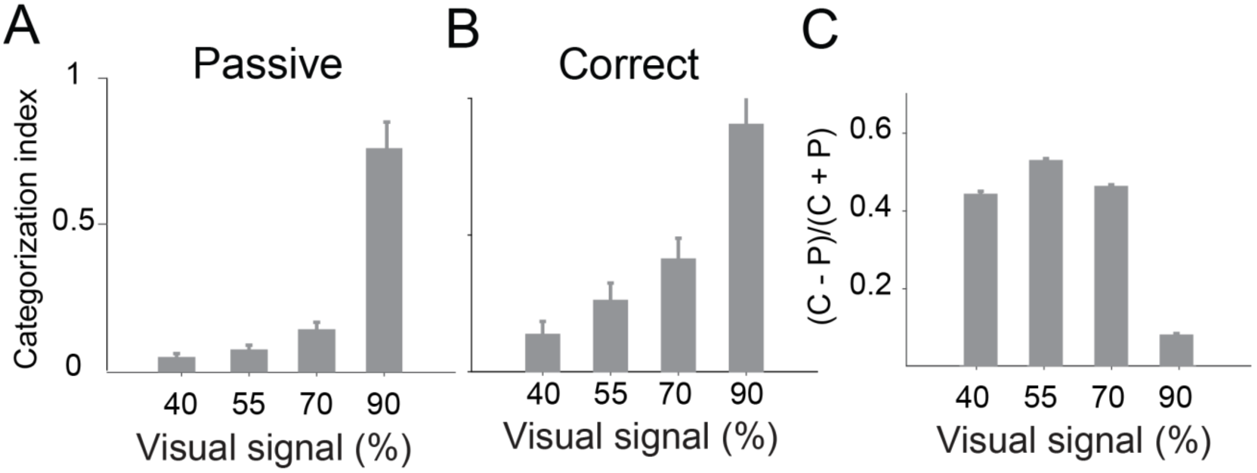

Figure S5. Categorization index in highly selective neurons.

Bar graphs illustrate the CI during 150 to 350 ms after stimulus onset in passive (A) and correct (B) trials computed using highly selective neurons. A similar task-dependent category information coding was observed in highly selective neurons (C). Error bars represent the STD of bootstrap samples.
